# Will We Need a Novel Heuristic in Resectable Lung Cancer?: A Narrative Review

**DOI:** 10.3390/curroncol33050245

**Published:** 2026-04-25

**Authors:** Lorenzo Gherzi, Marco Alifano

**Affiliations:** Thoracic Surgery Department, Hôpital Cochin, APHP Centre, University of Paris Cité, 75014 Paris, France; lorenzo.gherzi@aphp.fr

**Keywords:** lung cancer, heuristic, prognostic factors, outcome, survival

## Abstract

Introduction: The management of resectable non-small cell lung cancer has long relied on a relatively limited set of determinants, primarily anatomical resectability and pathological stage. Although these parameters remain central to therapeutic planning, accumulating clinical and translational evidence indicates that they do not fully explain variability in outcomes observed after lung cancer surgery. The primary aim of this review is to evaluate whether current evidence supports the need for a novel heuristic framework in resectable NSCLC. Secondary aims are to examine how host-related, clinical, and data-driven factors contribute to prognosis and treatment selection beyond conventional staging systems. Methods: This review integrates evidence from clinical studies, national registries, and translational analyses to examine how these dimensions contribute to prognosis and treatment selection. Results: Over the past two decades, advances in surgical techniques, perioperative management, systemic therapies, and large-scale clinical databases have revealed additional determinants of prognosis beyond tumor burden, including physiological reserve, nutritional condition, systemic inflammatory state, comorbidities, and socioeconomic environment. Developments in multimodal strategies and minimally invasive surgery have reshaped the therapeutic landscape. Data-driven approaches have identified clinically meaningful subgroups not captured by conventional staging systems. Conclusions: A heuristic framework integrating tumor biology, patient characteristics, and treatment context may better reflect the complexity of contemporary thoracic oncology practice.

## Simple Summary

Clinical decisions in operable lung cancer have traditionally relied on a limited number of criteria, mainly tumor stage and anatomical resectability. However, advances in surgery, systemic therapies, perioperative care, and clinical research have considerably expanded the knowledge available to clinicians. While this progress has improved treatment possibilities, it has also increased the complexity of clinical decision-making. In such contexts, physicians often rely on practical decision rules, known as heuristics, which help simplify complex information and support consistent choices in everyday practice. Growing evidence shows that outcomes after lung cancer surgery depend not only on tumor characteristics but also on patient-related factors such as nutritional status, physiological reserve, systemic inflammation, and social context. This review discusses how integrating these diverse elements may support the development of a new heuristic framework to guide treatment decisions and better reflect the complexity of modern surgical thoracic oncology.

## 1. Introduction

Lung cancer is the second most frequently diagnosed malignancy worldwide and the single leading cause of cancer-related death [1]. In France, it accounts for approximately 50,000 new cases and 30,000 deaths annually [2]. Despite these sobering figures, the landscape of the disease has evolved substantially over recent decades. The epidemiological profile is shifting: incidence is rising among women and among never-smokers, the proportion of adenocarcinomas is increasing relative to squamous cell carcinomas, and the fraction of cases diagnosed at early, potentially resectable stages is growing, partly as a consequence of computed tomography-based screening programs. A larger proportion of patients is now asymptomatic at diagnosis, and therapeutic efficacy across all modalities, including surgical, systemic, and radiological treatments, has improved considerably [3].

In terms of surgical outcomes, real-world data from the French nationwide EPITHOR database illustrate remarkable progress. Five-year survival in the overall lung cancer population remains limited at around 20%, yet among operated patients, it now approaches 60%, compared with about 40% two decades earlier [2,3]. Roughly one quarter of patients undergo surgery, whereas up to one third of cases could be operable. Multimodal treatment has become established from stage II onwards [4]. These changes create a setting in which historical rules remain essential but may no longer be sufficient on their own.

This progress has rendered the disease more complex to manage. The way diseases are known and conceptualized evolves alongside the diseases themselves. Improvements in biological understanding—encompassing tumor genetics, immune microenvironment, tumor-host interactions—have produced new therapeutic targets and transformed the multimodal treatment algorithm [3,5,6]. These advances demand a commensurate evolution in the conceptual tools that clinicians use to make decisions, particularly in the surgical arena. What kind of evolution do we need?

A heuristic, in cognitive and operational terms, is a method or empirical rule used to solve problems or reach decisions rapidly when optimal solutions are difficult or im-possible to determine [7]. Unlike algorithmic approaches, a heuristic does not guarantee the best available solution on the basis of the whole available knowledge, but typically provides a sufficiently good answer, quickly and with limited cognitive resources. Its defining characteristics include grounding in experience or intuition, efficiency in complex or uncertain environments, and susceptibility to systematic biases or errors. In clinical medicine, heuristics are unavoidable, they underpin triage, prognostication, and treatment allocation in every specialty. In thoracic oncology, the traditional heuristics that have governed surgical indications and perioperative management are facing unprecedented challenges from both the growing complexity of patient presentations and the expanding body of evidence that confounds classical assumptions.

The clinical profile of patients referred for surgery is no longer the same as in previous decades [3,5]. As the disease evolves and its biological understanding deepens, the question is no longer limited to whether a tumor can be removed. It increasingly concerns how resectability should be interpreted in patients whose outcomes are also influenced by other factors, including nutritional status, body composition, inflammatory profile, anthropometric phenotype, social deprivation, and metabolic heterogeneity [4,6,8]. The accumulation of such information raises the possibility that resectable lung cancer may need to be approached through a more integrative framework, capable of linking classical and novel surgical standards with a deeper understanding of host–tumor interactions.

**Figure 1:**
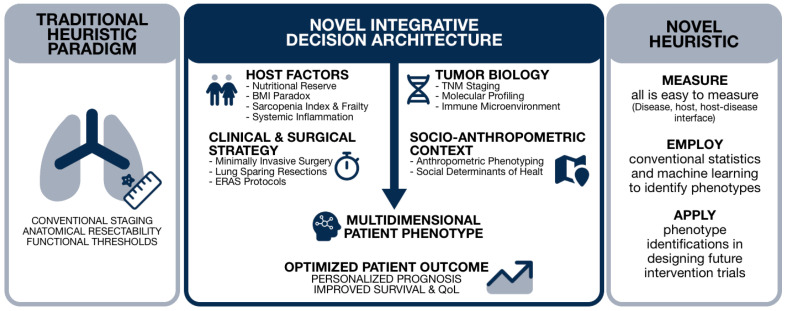
Conceptual map of transition from a traditional paradigm to a heuristic decision architecture.

The present review argues that the field now requires a novel heuristic, one that integrates tumor characteristics, patient-level factors, socioeconomic determinants, and molecular biology into a coherent decision architecture for resectable lung cancer. This argument is developed across the domains of surgical indications, extent of resection, perioperative strategies, prognostic factor integration, and translational science (Figure 1).

## 2. Methods

This narrative review was structured around a comprehensive synthesis of clinical, epidemiological, and translational evidence pertaining to resectable non-small cell lung cancer. The literature was organized thematically to address:

(1) the evolving epidemiological context and its implications for surgical practice;
(2) the classical and evolving criteria for surgical indication and their place within modern decision architecture;
(3) evidence from randomized controlled trials and real-world data regarding extent of resection;
(4) perioperative care optimization through enhanced recovery protocols;
(5) prognostic factors beyond pathological staging, including inflammatory and nutritional biomarkers, body composition, anthropometric variables, and social deprivation;
(6) emerging data-driven and biological approaches to patient clustering and outcome prediction.

Particular attention is given to studies and concepts demonstrating that outcome after lung cancer surgery may be influenced not only by tumor extent and resection quality, but also by measurable host characteristics that have historically received less emphasis in routine surgical reasoning. A multidisciplinary perspective was adopted, reflecting the collegial nature of modern thoracic oncological decision-making. Data from national registries, phase III randomized controlled trials, institutional cohort studies, and translational analyses are synthesized to support the central argument, thus articulating a unified narrative regarding the need for a novel heuristic in resectable lung cancer.

## 3. From Indication Criteria to Decision Architecture

Any attempt to propose a new heuristic in resectable lung cancer must begin by reaffirming the traditional framework for surgical indication. The classical criteria for operative candidacy have rested on a stable set of principles: the absence of distant metastasis (with limited exceptions), the technical feasibility of complete resection encompassing the primary tumor and regional lymph nodes, preserved respiratory function compatible with the planned extent of pulmonary ablation, an acceptable operative risk accepted by the patient, and the endorsement of the decision by a multidisciplinary tumor board. Direct contiguous extension to adjacent structures, such as the chest wall, superior vena cava, or carina, does not constitute a contraindication to surgery, provided that such involvement can be clearly distinguished from hematogenous or lymphatic metastatic spread. These principles remain the structural foundation of modern thoracic oncology. This conceptual shift from classical indication criteria to a more integrative heuristic decision framework is summarized in Table 1.

A novel heuristic would not replace them. Rather, it would enrich the reasoning by helping clinicians interpret situations in which technically resectable disease is embedded in substantial biological, physiological, or social heterogeneity. This point becomes especially relevant in settings such as stage III N2 disease, where multimodal sequencing, case selection, and expert judgment are already central to treatment allocation. In these situations, decision-making is rarely governed by a single deterministic rule. It instead depends on a contextual synthesis of available evidence, anticipated treatment tolerance, and the probability that local control will translate into durable benefit.

The contemporary classification of resectability in stage III NSCLC reflects an important conceptual evolution. While T1–2 single-station non-bulky N2 disease is generally considered resectable and bulky or invasive N2 disease is regarded as unresectable, a substantial intermediate category of “potentially resectable” disease remains. This group includes patients with multi-station non-bulky N2 involvement or T4 tumors by invasion, for whom individual multidisciplinary evaluation is recommended. The concept of potentially resectable disease acknowledges that the traditional binary distinction between operable and inoperable cases is insufficient for a significant proportion of patients. In particular, in multiple-station N2 disease the simple number of involved lymph node stations cannot be regarded as the sole determinant of resectability. Likewise, bulky N2 disease, typically defined by a lymph node short-axis diameter greater than 2.5–3 cm, may still warrant individual discussion in highly selected patients, particularly within multidisciplinary treatment strategies or clinical trial settings [9,10,11].

## 4. Dimensions of a Novel Heuristic in Resectable Lung Cancer

### 4.1. Functional Evaluation: Beyond Rigid Thresholds

One of the clearest manifestations of the progressive shift toward heuristic reasoning in thoracic surgery is found in perioperative functional evaluation. Evidence-based physiological guidelines remain indispensable, yet real-world practice increasingly extends beyond standard spirometric thresholds. The American College of Chest Physicians (ACCP) algorithm for preoperative functional evaluation exemplifies a structured heuristic: it begins with the computation of predicted postoperative FEV1 and DLCO, escalates through stair-climb and shuttle-walk tests when values fall below 60% of predicted, and ultimately relies on cardiopulmonary exercise testing with VO_2_max thresholds to stratify patients into low, moderate, and high operative risk categories [12]. The algorithm implicitly acknowledges that actual risk is additionally modified by patient comorbidities, structural aspects of the operating center, process-level factors in complication management, and surgical access strategy, variables that resist full quantification. The question is therefore no longer only whether lung function satisfies minimum criteria, but also whether additional, easily measurable variables can better identify the patient’s true capacity to tolerate surgery and recover from it.

Sarcopenia represents one such variable. Cross-sectional imaging can quantify muscle-related parameters including cross-sectional Total Psoas Area (TPA), Total Muscle Area (TMA), and Total Parietal Muscle Area (TPMA, defined as TMA minus TPA), with or without exclusion of fatty infiltration based on CT attenuation Hounsfield unit thresholds. Findings demonstrated that all sarcopenia parameters carried significant prognostic information, with TMA after fat exclusion emerging as the best discriminating variable (*p* < 0.001). Right-sided pneumonectomy and sarcopenia were independently associated with all three short-term outcome measures (postpneumonectomy respiratory failure, ARDS, and mortality) while the Charlson Comorbidity Index independently predicted acute respiratory failure alone [13,14,15]. The importance of these results lies not only in their statistical significance, but in the fact that they derive from routine pre-operative imaging and therefore offer a practical, immediately applicable route toward more refined perioperative risk stratification.

A further refinement in perioperative risk prediction is provided by the normalized pulmonary artery diameter (nPAD), defined as the crude pulmonary artery diameter divided by the square root of height. This index, derived from routine preoperative CT imaging, has been shown to be associated with the risk of postoperative respiratory failure, acute respiratory distress syndrome, and mortality after pneumonectomy. In this context, nPAD appears to provide complementary prognostic information when interpreted alongside traditional functional parameters such as predicted postoperative FEV1 and patient body habitus [16,17,18]. These findings illustrate how clinically meaningful predictors may emerge from familiar variables when they are reformulated in a more biologically coherent way. Such reinterpretations exemplify the type of pragmatic reasoning underlying a novel heuristic: a simple computational index, obtainable without additional testing, that can enrich perioperative risk stratification beyond conventional spirometric and functional assessments.

### 4.2. A Simple and Unexpected Heuristic: Nutritional Status and the Lung Cancer Paradox

Among the most counterintuitive findings to emerge from large-scale surgical data is the consistent demonstration that body mass index (BMI) exerts a paradoxical influence on postoperative outcomes in NSCLC—a phenomenon that has come to be termed the “lung cancer paradox” [4]. In patients undergoing pulmonary lobectomy for cancer, BMI was independently associated with operative mortality in a non-linear fashion. Specifically, underweight patients (BMI < 18.5 kg/m^2^) were found to have an increased risk of operative mortality compared with normal-weight patients, whereas overweight (BMI 25–30 kg/m^2^) and obese patients (BMI > 30 kg/m^2^) showed a lower risk of operative death. Similarly, pulmonary complications were significantly more frequent in underweight patients and less frequent in overweight patients [19,20,21,22].

These observations are difficult to dismiss as simple artifacts. They challenge the intuitive assumption that excess adiposity uniformly increases surgical risk and force a reconsideration of the meaning of body mass in resectable lung cancer. If higher weight is associated with better outcome while severe leanness is consistently unfavorable, then body habitus is likely capturing more than excess adiposity: it may reflect nutritional reserve, systemic resilience, latent frailty, or some broader interaction between host metabolism and tumor biology. Such findings encourage clinicians to move away from an exclusively tumor-centered interpretation of prognosis. This heuristic, that a higher BMI is associated with better short-term surgical outcomes, is simple, rapidly applicable, and supported by large-scale data, making it operationally useful despite its apparent contradiction with general surgical dogma.

### 4.3. Perioperative Care: An Enhanced Recovery Heuristic

Interest in the patient himself, rather than in his disease and the surgical strategies used to remove it, formed the basis for the development of Enhanced Recovery After Surgery (ERAS) protocols. The evolution of perioperative care in thoracic surgery offers another example of how structured heuristics can improve clinical outcomes. ERAS represent a systematic attempt to organize perioperative management around a set of practical principles aimed at reducing surgical stress, accelerating recovery, and standardizing best practices across the perioperative pathway [23].

In lung resection surgery, ERAS programs typically involve coordinated interventions across all phases of care. Preoperative preparation emphasizes patient education, optimization of nutritional and metabolic status, and minimization of prolonged fasting. Intraoperative management prioritizes minimally invasive techniques when feasible, balanced analgesia strategies aimed at reducing opioid exposure, and rational chest drainage strategies. Postoperatively, early mobilization, early resumption of oral intake, careful pain control, and rapid withdrawal of unnecessary invasive devices form the core of recovery-oriented care.

The conceptual relevance of ERAS lies not only in its clinical results but also in its methodological structure. ERAS protocols function as institutional heuristics, translating complex perioperative knowledge into a coherent set of practical rules that can be consistently applied by multidisciplinary teams. Rather than relying solely on individualized decision-making for each step of care, ERAS frameworks provide structured guidance that simplifies complex perioperative processes while remaining grounded in evidence-based principles.

Studies evaluating ERAS pathways in lung cancer surgery have shown that comprehensive perioperative optimization can be associated with low postoperative mortality and favorable long-term outcomes following major pulmonary resections [23,24,25,26]. More broadly, ERAS illustrates how the deliberate organization of routine clinical practices into structured care pathways can enhance both the efficiency and the safety of thoracic surgical management. The philosophy underlying ERAS seems simple: avoid anything that is disproportionately invasive or even useless, and promote patients’ well-being, even during difficult periods in their lives.

### 4.4. The Extent of Resection: Evidence-Based Heuristics and the End of a Dogma

The question of optimal extent of pulmonary resection in early-stage NSCLC has already undergone a conceptual revolution over the past decade. Lobectomy has been considered the gold standard for decades, with segmentectomy restricted to patients with insufficient pulmonary reserve and pneumonectomy reserved for cases of unavoidable anatomical necessity. This hierarchy constituted one of the most durable and widely accepted heuristics in thoracic oncology.

It was challenged decisively by the results of the JCOG0802/WJOG4607L trial, a multicenter, open-label, randomized, non-inferiority phase III study enrolling patients with peripheral NSCLC of diameter 2 cm or less and a consolidation–tumor ratio greater than 0.5 [27]. The trial demonstrated that segmentectomy was not only non-inferior but superior to lobectomy in terms of overall survival for this specific subgroup of patients, while relapse-free survival remained comparable between the two surgical strategies.

Concurrent evidence from the randomized trial CALGB evaluated sublobar versus lobar resection for peripheral T1aN0 NSCLC (tumor size 2 cm or less) [28]. Disease-free survival was not significantly different between groups, and recurrence patterns were comparable. These converging data from phase III randomized trials across different continents firmly established segmentectomy as the new standard of care for peripheral NSCLC lesions of 2 cm or less, definitively overturning a decades-old surgical dogma.

Real-world validation was provided by analysis of the EPITHOR French Registry encompassing patients with stage c-IA lung carcinoma [29]. In this population-based cohort, segmentectomy and lobectomy showed comparable adjusted long-term outcomes, while wedge resection was associated with significantly poorer survival.

However, this development invites important nuance. A new standard should not automatically be extrapolated to all patients with stage I disease. The subgroup in which superiority of segmentectomy was demonstrated is highly specific: peripheral lesions, diameter 2 cm or less, solid or predominantly solid consolidation–tumor ratio. As surgical indications evolve, the burden of accurate phenotypic selection becomes greater, not smaller. A modern heuristic is therefore necessary not because standards have disappeared, but because standards now require more precise interpretation of the population to which they truly apply.

### 4.5. Prognosis Beyond TNM: The Long-Term Impact of BMI on Survival

The prognostic impact of BMI extends well beyond the perioperative period and exerts a significant influence on long-term survival after pulmonary resection. Analyses of large nationwide surgical databases have shown that body mass index is independently associated with overall survival after lung cancer surgery, even after adjustment for major clinical and oncologic variables including age, comorbidities, performance status, extent of resection, histology, and pathological stage [4]. In these analyses, underweight patients consistently show worse long-term survival, whereas overweight and obese patients have better outcomes than those with normal BMI. This survival advantage associated with higher BMI persists across different patient subgroups. Furthermore, among obese patients, a gradient effect has also been observed, with progressively higher BMI associated with progressively improved survival.

A potential objection to the apparent protective effect of obesity is reverse causation bias, whereby lower BMI at the time of surgery might simply reflect underlying disease severity or cancer-related weight loss. This possibility has been specifically examined by comparing body mass index measured before cancer onset with BMI recorded at the time of surgery [30]. These analyses showed that both pre-disease BMI and preoperative BMI are independently associated with survival after lung cancer resection. Patients who had higher BMI before the onset of disease demonstrated better long-term survival, and weight loss prior to surgery was associated with worse outcomes, whereas weight stability or weight gain was linked to improved prognosis. Taken together, these findings suggest that the association between higher BMI and improved survival cannot be explained solely by reverse causation and support the hypothesis that adiposity may exert a genuine biological influence on outcomes in resectable lung cancer.

Concurrent evidence from other large datasets reinforces these observations. Analyses of patients undergoing resections for lung cancer have shown that both body mass index and objective measures of skeletal muscle mass, such as total psoas area, independently predict long-term survival [31]. Large national registry studies have similarly confirmed that preoperative BMI is associated with postoperative outcomes after complete resection for NSCLC [32]. Comparable findings have also been reported in cohorts of patients treated for locally advanced disease, where obesity has been associated with improved long-term survival following definitive treatment [33].

### 4.6. Extensions of the Lung Cancer Paradox

An unexpected extension of the lung cancer paradox was provided by the demonstration that not only weight but also height independently predicts long-term survival after pulmonary resection for NSCLC. Greater height was associated with progressively improved overall survival, independently of BMI [34,35,36]. This protective effect appears consistent across BMI categories, age groups, and clinical subgroups, suggesting that height represents a previously underappreciated host characteristic contributing to prognosis after lung cancer surgery. Although the magnitude of this effect is modest, it is reproducible across sensitivity analyses and indicates that anthropometric factors beyond BMI may carry clinically relevant prognostic information.

Height may therefore represent an additional dimension of the lung cancer paradox, potentially reflecting developmental, constitutional, or physiological reserve-related characteristics not adequately captured by BMI alone. While height is rarely considered in thoracic oncology decision-making beyond body surface calculations, its association with survival suggests that morphometric characteristics may contain prognostic signals that remain partly hidden within conventional indices.

This observation also highlights a broader methodological issue: BMI may not be the most informative way to represent the relationship between body morphology and outcome. If greater height and, possibly, greater weight are independently associated with improved survival, then BMI would inevitably compress two distinct biological dimensions into a single ratio. The prognostic signal attributed to BMI might therefore partly reflect underlying anthropometric features that deserve to be considered separately.

To address this limitation, refined morphometric indices have been developed to disentangle the contributions of weight and height [37]. One such approach introduced the Height-Normalized Weight (HNW), defined as the observed body weight divided by the mean weight expected for a given centimeter of height, calculated in sex-specific terms and stratified into quartiles. Height itself was similarly categorized into sex-specific quartiles (SH), and the two parameters were combined to create a composite SH/HNW index capturing both dimensions simultaneously. In multivariable analyses adjusted for major clinical and oncologic variables, this composite index demonstrated a clear gradient of prognostic discrimination, with progressively improved survival across increasing quartiles. Importantly, this association persisted across pathological stages and even within subgroups of patients classified identically by BMI, indicating that the combined morphometric index provides additional prognostic information beyond BMI alone.

Beyond its statistical performance, the relevance of this approach is conceptual. It illustrates that clinically meaningful insights may arise not only from introducing new variables, but also from reorganizing familiar variables according to a more biologically coherent framework. In this sense, anthropometry becomes a useful example of the heuristic principle underlying the broader argument of this work: simple measurements derived from routine clinical data may acquire greater predictive value when interpreted through alternative, biologically grounded combinations.

### 4.7. Prognostic Factors Beyond Stage: Inflammation, Nutrition, and the Tumor Immune Microenvironment

Other host-related biological factors provide additional prognostic information and can refine risk assessment beyond tumor burden and resection completeness.

Systemic inflammatory and nutritional markers represent one important category of such determinants. Composite indices incorporating hematologic and nutritional parameters, such as the HALP score (Hemoglobin × Albumin × Lymphocytes/Platelets), have been shown to stratify survival after lung cancer surgery and to provide prognostic discrimination within pathological stage groups [38].

A complementary dimension is provided by the interaction between systemic inflammation, nutritional status, and the tumor immune microenvironment. Combined analyses of circulating inflammatory markers, nutritional indicators, and intratumoral immune cell infiltration have demonstrated that these host-related factors independently influence survival after resection for NSCLC [39,40]. Together, these findings suggest that the integration of systemic host biology with tumor characteristics represents an additional heuristic framework for understanding prognosis beyond conventional staging alone.

### 4.8. Building a Prognostic Score: Integrating Disease and Patient Characteristics

The accumulation of evidence regarding multidimensional prognostic factors naturally raises the question of whether these can be integrated into a comprehensive and clinically validated prognostic score. This question is addressed through the development and validation of sex-specific prognostic nomograms for resectable NSCLC [41,42,43], by using the nation-wide French database EPITHOR.

In sex-specific multivariable models, age, BMI, height, comorbidities, WHO performance status, GOLD score, ASA class, surgical procedure, histological type, and pathological stage were all retained as independent predictors of survival. The final model demonstrated excellent discrimination and calibration in both development and validation sets, confirmed by calibration plots showing close agreement between predicted and observed 3- and 5-year survival probabilities. Kaplan–Meier analyses of predicted fifths of risk showed wide survival differences across all stages, with the lowest-risk fifth of stage I patients achieving near-plateau survival curves and the highest-risk fifth approaching the survival of stage II patients, a finding with direct implications for personalized adjuvant treatment allocation. These nomograms, made available as an open-access tool, operationalize the heuristic insight that prognosis after pulmonary resection is determined by a constellation of patient, disease, and procedural factors rather than by stage alone.

If multiple modest variables each contribute partial prognostic information, then one logical step is to integrate them rather than interpret them one at a time. This is where multidimensional phenotyping becomes particularly attractive. A multidimensional representation of risk, combining disease factors, patient factors, and their interface, may be closer to clinical reality than conventional linear stratification based on one or two dominant variables, and may help explain why patients with apparently similar tumors experience very different postoperative trajectories.

### 4.9. From Host-Tumor Interface to Multidimensional Phenotyping: Social Deprivation as an Independent Prognostic Determinant

The prognostic factors discussed above are largely biological or morphometric in nature. However, emerging evidence indicates that socioeconomic determinants—factors external to the individual’s biology yet indelibly shaping health trajectories—are equally important determinants of outcome after pulmonary resection [8,44]. This observation expands the host-tumor model beyond physiology and metabolism to include environmental and structural determinants of outcome. A modern heuristic must therefore account for the fact that patients do not experience cancer in isolation from their social context.

The French Deprivation Index (FDEP), a geographical indicator of social deprivation based on the patient’s department of residence, has been shown to be independently associated with long-term survival after pulmonary resection for NSCLC [45]. In analyses of the nationwide EPITHOR cohort, patients operated on in the most socially deprived areas experienced significantly poorer long-term survival compared with those from the least deprived areas, with a progressive gradient of risk across increasing levels of deprivation.

This association was also observed in an independent single institution cohort, where social deprivation of the area where patients live remained associated with survival in both unadjusted and adjusted analyses. Importantly, the impact of deprivation was evident across pathological stage categories, suggesting that socioeconomic context may influence outcomes beyond tumor-related characteristics alone.

This finding has profound implications: social determinants of health exert an independent influence on oncological outcome comparable in magnitude to differences in pathological stage, and they are in principle modifiable at the policy level. A clinically actionable heuristic that ignores socioeconomic context therefore systematically misjudges prognosis for a substantial fraction of patients.

### 4.10. Multimodal Management: Perioperative Systemic Therapies

Since stage II and beyond, the concept of multimodal management has been firmly established. The landscape of perioperative systemic therapy has been substantially transformed by the introduction of targeted therapies and immunotherapies in both neoadjuvant and adjuvant settings. The ADAURA trial demonstrated that adjuvant Osimertinib significantly im-proves overall survival in patients with resected EGFR-mutated NSCLC, establishing targeted therapy as the standard of care for this molecular subgroup across stages II to IIIA [46].

The CheckMate 816 trial evaluated neoadjuvant Nivolumab plus chemotherapy versus chemotherapy alone in patients with stage IB to IIIA resectable NSCLC, reporting five-year overall survival of 65% versus 55% in favor of the combination regimen in an open-label phase 3 trial [47]. Other trials—IMPOWER 030 (Atezolizumab plus chemotherapy, stages II–IIIB), KEYNOTE 671 (Pembrolizumab plus chemotherapy, stages IIB–IIIA), and AEGEAN (durvalumab plus chemotherapy, stages IIA–IIIB)—are further defining the neoadjuvant immunotherapy landscape across different stages and check-point inhibitor platforms [48,49,50].

Whether all eligible patients should receive neoadjuvant immunotherapy regardless of tumor biology and individual patient characteristics, or whether the benefit is concentrated in phenotypic subgroups, represents a critical unresolved question that necessitates more refined prognostic discrimination than current staging alone can provide. The same principle applies to intensification in early-stage disease: if some patients derive a meaningful survival gain from more complex perioperative strategies, this does not imply that all should receive such treatment. The challenge is to determine which patients are likely to benefit sufficiently to justify additional burden, toxicity, or complexity: a determination that cannot rely on stage alone, and that calls directly for the kind of integrative heuristic this review proposes. The above-described score could provide ideas on how construct future trials of perioperative interventions aiming at evaluating benefits on an almost-individual basis.

## 5. Why a Novel Heuristic in Resectable Lung Cancer Is Now Plausible?

Taken together, the evidence reviewed above supports the plausibility of a novel heuristic in resectable lung cancer. Such a heuristic would not consist of a single score or a single algorithm. It would instead represent a mode of clinical reasoning that accepts complexity while preserving usability—a decision architecture connecting standard oncologic and surgical principles with a structured recognition that prognosis and treatment tolerance are shaped by host-related, anthropometric, nutritional, metabolic, and social determinants.

This framework is attractive for several reasons. First, many of the relevant variables are easily accessible in routine practice: BMI, height, weight, preoperative blood samples, CT-derived muscle area, and a geographical deprivation index require no additional investigations. Second, they may help explain why patients with apparently similar tumors experience very different postoperative trajectories. Third, they may support more precise selection for extent of resection, perioperative optimization, multimodal treatment, and follow-up intensity. Fourth, multidimensional phenotyping creates a conceptual bridge between classical thoracic surgery and a more integrated form of thoracic oncology in which the patient phenotype is considered alongside the tumor phenotype.

The broader concept of P4 surgery—personalized, predictive, preventive, and participatory—provides the philosophical framework for this translational research agenda [51]. The ambition to expand the horizons of surgical research toward a precision medicine paradigm in thoracic oncology requires exactly the kind of heuristic renovation that the evidence reviewed in this article supports. As Galileo observed, the scientific ambition must be to measure what is measurable and to render measurable what is not yet so.

### The Advent of Machine Learning and Artificial Intelligence

The foregoing sections have addressed host and tumor-level heuristics based on simple concepts and traditional statistics. A complementary strategy consists of integrating multiple modest predictors rather than interpreting them individually. If several variables each contribute partial prognostic information, a logical next step is to combine them within a multidimensional framework in the era on artificial intelligence. This is where multidimensional phenotyping becomes particularly attractive, as it allows disease-related factors, patient characteristics, and elements of the host-tumor interface to be considered simultaneously rather than in isolation.

Unsupervised machine learning methods provide a practical framework for this approach. Algorithms such as K-means clustering group similar observations into “un-labeled” clusters within a multidimensional space. This framework contrasts with traditional “labelled” analyses, in which predefined variables or markers are examined in-dividually according to a prior hypothesis. In thoracic oncology, where prognosis reflects the interaction of multiple overlapping dimensions, clustering offers a way to capture structures that may remain invisible within conventional labeled approaches [52].

The conceptual value of this strategy is illustrated by work in tumor biology, where transcriptomic analyses of glucose metabolism in lung adenocarcinoma have identified distinct metabolic phenotypes associated with different clinical outcomes [53]. These findings emerged from the exploration of unlabeled biological data, rather than from single-marker labeled reasoning, demonstrating how clinically relevant heterogeneity may arise from latent groupings within complex datasets.

A similar logic can be applied at the patient level. Using clustering methods capable of integrating both numerical and categorical variables, analyses of surgical NSCLC cohorts have identified groups of patients sharing distinct clinical phenotypes and survival trajectories [52]. In these models, variables such as sex, age, anthropometric characteristics, functional status, pulmonary function, comorbidities, and social deprivation con-tribute jointly to cluster formation. The resulting patient groups show significantly different overall survival patterns, both in the whole population and among early-stage disease, suggesting that phenotypic clustering can reveal prognostically relevant structures that remain hidden when variables are considered individually within conventional labeled frameworks.

Beyond unsupervised approaches, recent advances in artificial intelligence have expanded the potential for integrating imaging, clinical, and biological data into unified predictive models. Radiomics and deep learning techniques applied to CT and PET imaging have demonstrated the ability to extract high-dimensional quantitative features that may correlate with tumor biology, treatment response, and survival outcomes [54,55]. These approaches enable the identification of latent patterns not visible to human interpretation and support the development of predictive models that integrate imaging phenotypes with clinical and molecular variables.

More broadly, artificial intelligence is increasingly viewed not as a replacement for clinical reasoning, but as an extension of it. In thoracic oncology, AI-driven models may assist in risk stratification, treatment selection, and outcome prediction, particularly in complex scenarios where multiple competing factors must be considered simultaneously. Importantly, these systems remain dependent on the quality, structure, and representativeness of the data on which they are trained, and their integration into clinical practice requires careful validation and interpretability [56]. In this context, artificial intelligence can be understood as a technological embodiment of the heuristic approach proposed in this review: a means of synthesizing multidimensional information into clinically meaningful patterns that support, rather than replace, expert decision-making.

## 6. Conclusions

Resectable lung cancer has entered an era in which traditional surgical standards remain indispensable but are increasingly insufficient as the sole framework for decision-making. Anatomical resectability, pathological stage, complete resection, pulmonary reserve, and multidisciplinary discussion still define the basis of operative management. However, accumulating evidence indicates that host-related factors—including sarcopenia, inflammatory and nutritional state, anthropometric phenotype, body composition, pulmonary vascular metrics, and social deprivation—can refine prognosis beyond stage alone and, in doing so, demand a more integrative approach to clinical reasoning.

At the same time, changes in the evidence surrounding segmentectomy, perioperative treatment intensification, and multidimensional phenotyping suggest that treatment selection should progressively move toward a more integrated interpretation of the patient and the tumor. The central implication is not that classical criteria should be discarded, but that they should be embedded within a broader and more coherent decision architecture. The most useful future framework may therefore be one that combines surgical principles with a structured reading of host-tumor complexity, allowing decision-making to become both more individualized and more biologically meaningful.

In this sense, the answer to the question posed in the title is likely yes. Resectable lung cancer may indeed require a novel heuristic, not because established standards have failed, but because the field now possesses a richer understanding of biological and clinical heterogeneity than those standards were originally designed to handle. The data exist, the methodological tools are available, and the clinical urgency is real.

## Figures and Tables

**Table 1:** From classical indication criteria to a heuristic decision framework in resectable NSCLC.

Domain	Classical Framework	Heuristic Framework (Proposed)	Clinical Implication
Oncological status	Absence of distant metastasis	Integration of tumor burden with biological aggressiveness (molecular profile, immune context)	Better selection of patients likely to benefit from surgery within multimodal strategies
Tumor resectability	Technical feasibility of R0 resection	Contextual interpretation of resectability within patient and tumor heterogeneity	Moves from binary (resectable/unresectable) to probabilistic reasoning
Nodal disease	Anatomical classification (N0–N2, bulky vs. non-bulky)	Integration of nodal pattern with treatment response and systemic disease dynamics	Supports individualized multimodal sequencing
Functional status	Spirometry-based thresholds (FEV1, DLCO, VO_2_max)	Integration with sarcopenia, body composition, and imaging-derived metrics (e.g., nPAD)	More accurate prediction of perioperative risk
Operative risk	Global clinical assessment (age, comorbidities, ASA, PS)	Multidimensional risk including frailty, inflammation, nutrition, and metabolic status	Refines perioperative decision-making
Anthropometry	BMI considered marginally or descriptively	Integration of BMI, height, and composite indices (e.g., HNW)	Captures prognostic information beyond traditional metrics
Host biology	Limited consideration	Integration of systemic inflammation, nutritional markers, immune status	Links host condition to oncologic outcomes
Socioeconomic factors	Rarely considered	Inclusion of social deprivation and environmental context	Accounts for non-biological determinants of survival
Decision process	Rule-based, guideline-driven	Heuristic, integrative, and context-dependent reasoning	Reflects real-world complexity
Data integration	Single-variable or linear models	Multidimensional phenotyping and clustering approaches	Identifies clinically meaningful subgroups beyond staging

## Data Availability

No new data were created or analyzed in this study. Data sharing is not applicable to this article.
